# Research on Supportive Psychosocial, Drug Treatment, and Health Education Intervention and Health Management Model of Community-Residing Elderly Adults With Late Life Depression in Liaoning Province: A Protocol

**DOI:** 10.3389/fpsyt.2020.00267

**Published:** 2020-04-03

**Authors:** Li Duan, Xiaojun Shao, Chunfeng Fu, Chunsheng Tian, Gang Zhu

**Affiliations:** ^1^Department of Psychiatry, The First Affiliated Hospital of China Medical University, Shenyang, China; ^2^Central Laboratory, The First Affiliated Hospital of China Medical University, Shenyang, China

**Keywords:** late life depression, community, risk factor, supportive PDH intervention, health management

## Abstract

**Background:**

Late life depression (LLD), a common mental disorder, has become an increasingly acute public health concern with a quickly expanding geriatric population worldwide. To our knowledge, however, the incidence of LLD in northern cities in China has not been empirically investigated, and many elderly people with depressive moods and mild depressive symptoms have not been given sufficient attention.

**Methods/Design:**

This is a multi-stage and prospective study. The first stage is a cross-sectional study, investigating the epidemiological characteristics of LLD in northern China and exploring the biological, psychological, and social risk factors for developing LLD based on a set of questionnaires from 6,800 community-residing elderly adults. The second stage involves statistical analysis, by constructing a risk factor model for LLD and analyzing their direct and indirect functional routes on the basis of structural equation modeling. The third stage is an experimental study a total of 60 elderly patients with LLD and their principle caregivers will be randomly assigned to control and trial groups. The trial group patients and caregivers will undergo supportive psychosocial, drug treatment, and health education (PDH) intervention, whereas the control group patients and caregivers will be treated routinely (treatment as routine, TAR, which includes drug treatment and health education). At the end of the intervention, depressive symptoms, quality of life, and the social and cognitive functioning of the patients in the two groups will be respectively assessed at a baseline and after 6, 9, and 12 months post-intervention by employing scales and questionnaires to analyze the effectiveness of the supportive PDH intervention measures in comparison with TAR. Ultimately, a supportive PDH intervention and health management model will be obtained by combining PDH intervention with mental health institutions, community health services, and aging families as the main line.

**Discussion:**

This study will provide strong and suitable evidence for enhancing the integrated supportive PDH intervention and health management model of LLD patients among community-dwelling residents.

**Ethics:**

This study has been approved by the Ethics and Research Committee of the First Affiliated Hospital of China Medical University (approval No. [2019] 2019-312-2).

## Introduction

Late life depression (LLD) is complex, burdensome, and difficult to treat. Specifically, it refers to elderly people who, for the first time in their lives, meet criteria for major depressive disorder (MDD) or display clinically significant depressive symptoms such as functional disability, cognitive decline, increased risk of medical comorbidity (e.g., co-dementia), and even suicide in severe cases (1–4). Meanwhile, MDD in the elderly, as defined by the Diagnostic and Statistical Manual of Mental Disorders, Fourth Edition, Text Revision (DSM-IV TR), has been a growing public health concern, and it is projected to be the second leading cause of disease by the year 2020 (5, 6). Epidemiological surveys reported the prevalence of MDD in elderly adults in different countries, such as the USA at 16% (7), England at 8.7% (8), and India at 24.1% (9), with considerable regional variation. Additionally, the results of a meta-analysis based on 28 studies and 76,432 subjects showed that the point, 12-month, and lifetime overall prevalence of MDD in elderly adults was, respectively, 2.7%, 2.3%, and 2.8% in China (10).

Evidence shows that successful antidepressant treatment is one of the most effective ways of reducing disability, preventing morbidity, and improving quality of life (QOL) in older patients (11). However, the response rate to an antidepressant trial with adequate dose and duration is often inadequate, and can be as low as 30%~40% in LLD patients (12). Moreover, studies have found that it is difficult to improve the cognition and personality traits of elderly patients with pharmacotherapy alone, and a number of adverse events (e.g., cardiovascular, gastrointestinal, and anticholinergic complications) are often associated with antidepressant use. Frequently, this leads to discontinuation, which contributes to a high recurrence rate and mortality (13, 14). Thus, various forms of non-pharmacological therapies with negligible side effects are being embraced by patients, such as meditation, motor therapy, cognitive behavioral therapy, interpersonal psychotherapy, reminiscence therapy, alternative therapies, and group or educational activities (15–19). These treatments are tailored to the abilities of elderly patients and target multiple contributing factors with the aim of alleviating loneliness and comorbid anxiety, enhancing social support, and improving QOL.

In Western countries in the 1970s, health management, an important topic in the field of healthcare, developed rapidly and was successfully implemented for chronic conditions such as hypertension, diabetes mellitus, and chronic obstructive pulmonary disease (20–22). By combining information technology, geriatric medicine, and community medicine, health management has been used to strengthen the management of chronic diseases in the elderly, improve their lifestyle, and reduce mortality rates from disease (23). In China, relevant documents promulgated by the Chinese State Council clearly stated that health management is a fundamental way to improve the health quality of the elderly, extend life expectancy, and achieve healthy aging. However, there are few studies on community-based health management of patients with LLD, even though it is imperative to develop health management services for the elderly in the field of gerontology.

According to literature review and analysis, we found that there are four main deficiencies of the existing research. First, due to a large elderly population base, rapid aging growth, uneven distribution of mental health resources, and insufficient public attention to the mental health of the elderly, under-recognition and under-treatment of depression appears to be severe in Liaoning which ranked first among 19 provinces and cities in terms of the proportion of the elderly population aged 65 and above in 2016. Second, research on interventions for LLD is limited. Previous studies have been mostly based on pharmacotherapy, psychosocial intervention, health education, or a combination of any two of these, but none, to our knowledge, have been conducted combining all aspects together. Third, research on the risk factors of LLD has been limited, and the methods used have not been sufficiently systematic. Previous studies were limited to investigating risk factors (24–26), but lacked any discussion of the internal relations among the various factors. As such, they were limited with regard to providing guidance based on their results and conclusions for the treatment and intervention of diseases. Fourth, there is a lack of systematization and wholeness in research on the intervention of LLD. Previous studies mostly focused on tests of intervention methods. Due to impoverished intervention resources, objectives, and effects, and intervention objects and their background environment, intervention measures often have poor extensibility and cannot be repeated to verify research results. Here, we will comprehensively analyze the risk factors leading to LLD in Liaoning province, and construct integrated supportive PDH intervention and health management model of LLD patients among community-dwelling residents, aiming to provide scientific evidence for the implementation of interventions and health management, as well as formulation of relative health policies.

## Objectives

This study is designed to comprehensively assess the overall community mental health resources available to LLD patients, along with the prevalence, risk factors, and prevention and treatment status of LLD in Liaoning. By exploring and constructing the integration of psychosocial interventions, medical care, and health education with psychiatric hospitals, community health services (CHS), and aging families, this study can provide a reference for the practice of supportive intervention and health management, as well as guidance for formulating health policies in Liaoning. The aims of this study are as follows:

1. Analyze the epidemiological characteristics of LLD in Liaoning province.
2. Construct a risk factor model for LLD in Liaoning.
3. Conduct supportive psychosocial, drug treatment, and health education (PDH) interventions alongside treatment as routine (TAR) in a trial group and control group, respectively, to compare their effectiveness with regard to patients and their caregivers.
4. Establish a supportive PDH intervention and health management (SIHM) model for elderly patients with depression according to the local economic level, mental health resources, and demographics of Liaoning.

## Methods/Design

### Organizational Structure

Team members of the preliminary investigation will consist of six graduate students studying psychiatry, two clinical psychiatrists with experience in the fields of mental health, geriatrics, and medical psychology, and five CHS staff members from Liaoning province. Before implementing the questionnaire survey, graduate students and CHS staff will undergo unified training, to explain the specific purpose, content, significance, procedures, and principles of the investigation. Further, the team members will learn the definitions and scoring criteria of each item in the questionnaires and scales, communication skills for interacting with elderly patients and their caregivers, and possible problems and solutions that might be encountered. After sampling according to inclusion and exclusion criteria, the psychiatrists, accompanied by other team members, will enter selected communities, ask permission from community leaders, and complete the investigation in public activity areas or at elderly residences accompanied by the arranged community staff.

In addition, team members of the intervention will comprise graduate students in psychiatry, one clinical psychiatrist, and one psychologist employed to supervise and guide the intervention process. After team members work out a detailed intervention plan according to the purpose of the project and the characteristics of the research objects, the research team will organize experts in psychiatry, psychotherapeutics, social medicine, and geriatrics to review and approve the plan. Afterwards, the plan will be revised and improved based on expert advice before implementation. Then, in the course of carrying out the plan, the intervention team members will record any problems with the intervention process and immediately report back to the project leader for revisions before further implementation. The goal of such cycles is to ensure the effectiveness of the intervention.

### Quality Control

In order to ensure the quality of the preliminary investigation of this project, we formulated the following measures. First, there will be unified training for researchers before data collection to reduce bias caused by differences among researchers. Second, when carrying out the questionnaire, the environment will be as quiet as possible, so as to facilitate the emotional stability of the research subjects and avoid adverse effects from the internal and external environment. Third, when collecting the questionnaires after completion, missing items will be promptly identified (without violating the principle of voluntariness) to ensure the accuracy and integrity of the survey data. Finally, data obtained from the survey will be input into statistical software for analysis by two investigators working together. At the same time, before statistical processing, SPSS descriptive statistics will be used to verify the recorded data, and errors will be corrected.

Similarly, the following three measures were developed to ensure the quality of the intervention. First, members of the intervention team will regularly report the concrete content of the intervention measures and the implementation process to the project leader and clinical psychiatrists. Second, a clinical psychiatrist will be invited to evaluate, guide, and correct the accuracy of the intervention measures and methods, along with any problems with the intervention and suitable solutions. Third, contingency plans will be developed and imparted to researchers in case of any emergencies while executing intervention measures.

### Study Design

This is a multi-stage and prospective study. It includes the following three stages:

1. The first stage is a cross-sectional study, based on the baseline information from 6,800 community-dwelling elderly adults collected through a set of questionnaires. The aim of the first stage is to investigate the epidemiological characteristics of LLD in northern China, and to explore the biological, psychological, and social risk factors for developing LLD.
2. The second stage involves statistical analysis, with the aim of constructing a risk factor model for LLD and analyzing direct and indirect functional routes on the basis of structural equation modeling (SEM).
3. The third stage is an experimental study, comprising a 6-month supportive intervention period and a 6-month follow-up period. In order to ensure the participation of the subjects in this study and improve the effectiveness of the interventions, a total of 60 elderly patients with depression and their principle caregivers will be randomly and equally assigned to trial and control groups.

Trial group patients and caregivers will undergo supportive PDH interventions, and control group patients and caregivers will receive TAR. Subsequently, depressive symptoms, QOL, and the social and cognitive functioning of the patients in the two groups will be respectively assessed at a baseline and after 6, 9, and 12 months post-intervention by using scales and questionnaires to analyze the effectiveness of the supportive PDH intervention measures. Ultimately, an SIHM model will be constructed by combining PDH intervention with mental health institutions, CHS, and aging families as the main line.

### Participants

We plan to enroll the participants between December 2019 and December 2022. The goal is to recruit 6,800 community-residing elderly adults to complete the baseline investigation. Then, 60 LLD patients who meet more strict criteria, along with their principle caregivers, will receive 6 months of supportive PDH intervention followed by a 6-month follow-up period. The inclusion and exclusion criteria for all the respondents, patients with LLD, and caregivers are listed in **Table 1**.

**Table 1:** Inclusion and exclusion criteria for community-residing elderly adults in rural and urban cites/villages in Liaoning province.

Research subjects	Inclusion criteria	Exclusion criteria
6,800 community-residing elderly adults	aged 60 years old or above at the time of enrollment; permanent urban/rural community-dwelling residents (at least 5 years of residency) with urban/rural *hukou* status in Liaoning province; comprehension, reading, and writing skills to independently complete the questionnaires/scales or complete them with the assistance of the researchers without obstacles; voluntary participation and signed informed consent.	serious health concerns, such as acute infectious diseases, unstable cardiovascular disease, etc.; lifetime substance or alcohol dependence; high suicide risk; met criteria for dementia (major neurocognitive impairment in DSM-5); in the process of a depressive episode; former permanent urban/rural community-dwelling resident that has been away for 1 year or more.
60-LLD patients	aged 60 years old or above at the time of enrollment; permanent urban/rural community-dwelling residents (at least 5 years of residency) with urban/rural *hukou* status in Liaoning province; meet diagnostic criteria for MDD without psychotic features based on the Chinese version of MINI according to DSM-IV TR (the core diagnostic criteria of the symptomatology and course of disease are consistent with DSM-5). comprehension, reading, and writing skills to independently complete the questionnaires/scales or complete them with the assistance of the researchers without obstacles; voluntary participation and signed informed consent.	1) serious health concerns, such as acute infectious diseases, unstable cardiovascular disease, etc.; lifetime substance or alcohol dependence; high suicide risk; met criteria for dementia (major neurocognitive impairment in DSM-5); in the process of a depressive episode; former permanent urban/rural community-dwelling resident that has been away for 1 year or more.
60-principle caregivers	assumes most of the responsibility of caring for the patient (e.g. daily care, accompanying the patient to medical treatment, etc.) no communication barriers; voluntary participation and signed informed consent.	caregiver who receives remuneration; history of any psychiatric disorder; suffers from diagnosed organic or psychiatric disease (e.g., depression); refuses to provide authentic and reliable information to the research team.

LLD, late life depression; MINI, mini-international neuropsychiatric interview; DSM-IV TR, Diagnostic and Statistical Manual of Mental Disorders, Fourth Edition, Text Revision; MDD, major depressive disorder.

### Enrollment, Supportive PDH Intervention, and Follow-Up

1. The 6800 community-residing elderly adults will be screened and enrolled by the investigators according to the inclusion and exclusion criteria. To ensure the balance and representativeness of sample sources, we intend to adopt a multi-stage sampling method. At the first stage, four cites will be randomly selected in Liaoning province by randomized sortition. At the second stage, one district and one township will be randomly selected from each selected city. At the third stage, one urban community and one village community will be randomly selected from each selected district/township. At the fourth stage, elderly residents will be selected from each selected urban community and village community with a convenient sampling method.
2. The 60 community-residing elderly adults with LLD and their principle caregivers will be allocated to trial and control groups. In addition, we will ensure that there are no statistical differences between the two groups in term of the degree of depression, drug treatment programs, knowledge of mental health, geographic distribution, and other aspects before carrying out the interventions. The trial group of 30 elderly patients and their principle caregivers will receive the same content and frequency of individualized and group intervention for 6 months. By contrast, the 30 elderly patients in the control group and their principle caregivers will receive TAR, which are suitable for all MDD patients as routine. During this period, except for any additional treatment for comorbid physical diseases or short-lasting benzodiazepines for severe insomnia, other antipsychotic medications, systematic psychotherapy, and long-lasting benzodiazepines will not be allowed. The detailed content, forms, and methods of the supportive PDH intervention are listed in **Table 2**.

**Table 2:** Content, form and method of supportive PDH interventions.

	Supportive PDH interventions
Content	P: Supportive psychosocial intervention Supportive therapy, including the application of listening, sympathy, comfort, and guidance, constructive and instructional language, asking patients to actively communicate with themselves and intervention team members. Cognitive behavioral therapy, including the correction of abnormal cognition and behavior, and guiding patients to correctly face major stressful events in life or attitudes and treatment skills in the event of recurrence. Social skills training, including self-care skills training, interpersonal skills training (e.g., positive communication with family members, and active participation in related group activities). Mindfulness therapy, including breathing, mindfulness, and walking. Interest cultivation, including encouraging patients in the trial group to participate in outdoor sports therapy and entertainment therapy (e.g., painting, carpentry, clay, knitting, chess, card games, etc.), and cultivating personal interests. Group nostalgia therapy, including guiding patients to recall old classic songs, movies, important events, happy childhood events, and achievements made in life or at work. D: Supportive drug treatment Patients will be guided to use drugs strictly in accordance with a safe and standardized system. Principle caregivers will be normatively urged to provide supervision to improve the medication compliance of the patients. H: Supportive health education Mental health education, including symptoms, early signs of recurrence and preventive measures of LLD. Drug treatment health education, including knowledge about drugs (e.g., antidepressants), the importance of maintaining and taking medicine on time, the significance of pharmacotherapy for preventing recurrence and deterioration, and self-management skills for drug therapy. Lifestyle health education, including establishing a reasonable and balanced diet, good sleep hygiene, quitting smoking, limiting alcohol, and adhering to a moderate exercise regime.
Form	P: Supportive psychosocial intervention Rely on the platform of CHS to carry out group psychological intervention for patients. Rely on the family, and have intervention team members work with the principle caregivers to provide psychological counseling and personalized treatment for patients. H: Supportive health education Distribute manuals and pamphlets on the prevention and treatment of depression in the elderly. Regularly carry out group discussions and lectures for patients and their principle caregivers. Open a psychological consultation platform on the Internet. Cooperate with CHS to establish an LLD rehabilitation self-help group and organize group activities regularly. (group therapy will be conducted once a month, and family and individual therapy will be conducted at least twice a month)
Method	Make physical examination appointments or temporary on-site appointments with patients and their principle caregivers. Collect patient health information and establish health management files. Health assessment and prediction, with a detailed intervention plan. Pre-intervention training. The project leader and main group members will conduct unified training for follow-up CHS members. The main content of the training will pertain to symptoms and condition assessments, medication and efficacy observations, physical and mental health recording, interviewing skills, psychosocial intervention methods, and home visits and telephone follow-up skills. Intervention. The follow-up members should strictly follow the intervention outline formulated by the research group and adopt the supportive PDH method to complete the intervention. The project team will arrange fixed personnel to supervise and guide the members regularly. Overall, the intervention will last 6 months. Follow-up. In the first 3 months after the intervention, a monthly home visit and a telephone follow-up will be conducted. A telephone follow-up will be conducted every 2 weeks for the next 3 months, where the follow-up period will last 6 months. During each follow-up, it is necessary to evaluate the health management status of the patients and any intervention goals that have been achieved, while devising new goals based on the actual situation, and providing corresponding individual counseling according to the patients' mastery of relevant knowledge, skills, and personal needs.

Considering that Shenyang, the capital of Liaoning, has the second-largest elderly population in the province, a multi-stage sampling method will be applied during this phase as well. At the first stage, one district and one township will be randomly selected by randomized sortition in Shenyang. At the second stage, one urban community and one village community will be randomly selected from each selected district/township. At the third stage, with the help of local community health workers, 60 elderly patients with LLD who meet the inclusion criteria will be selected from each selected urban community and village community with a convenient sampling method.

**Figure 1:**
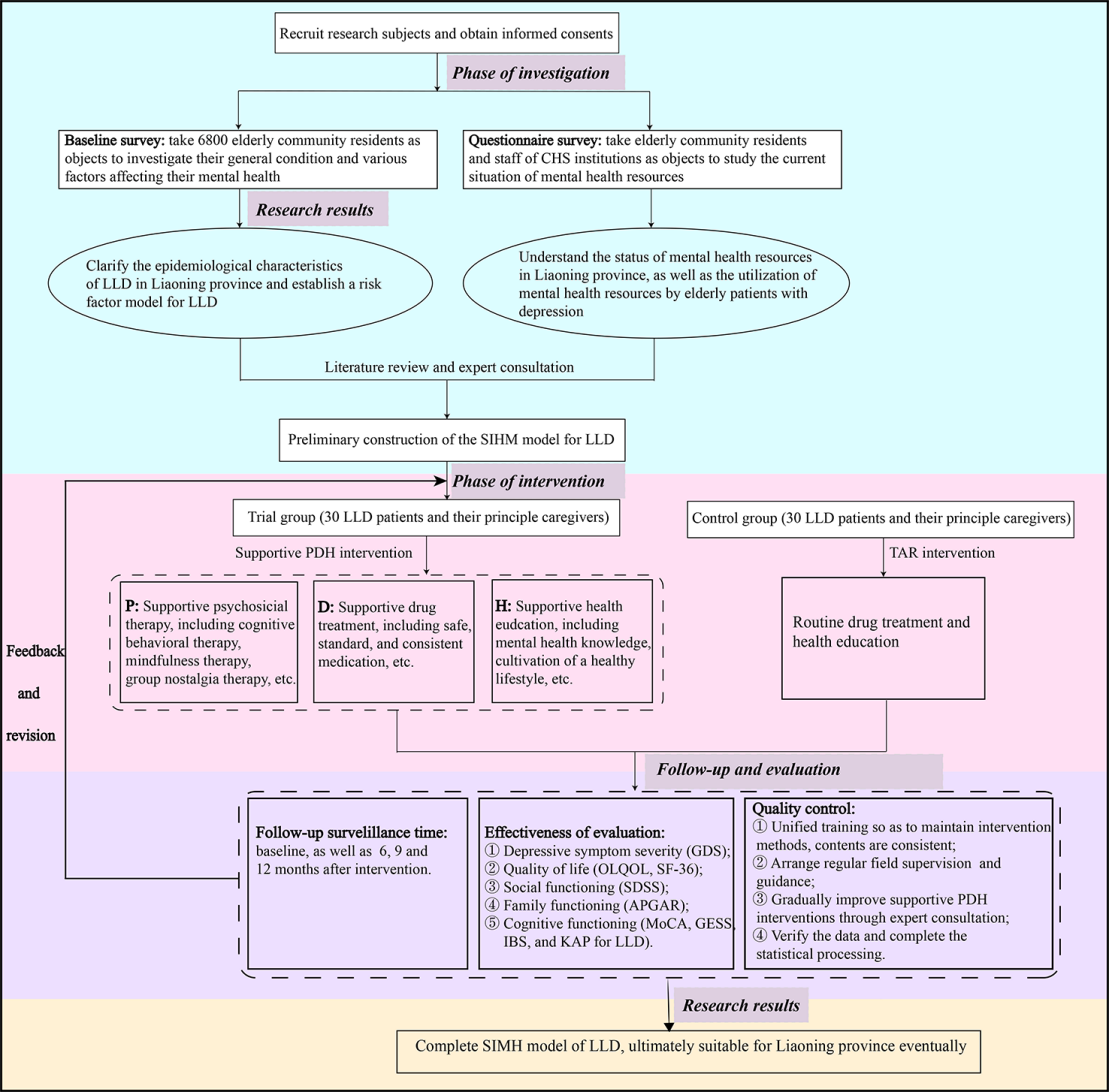
The research flowchart.

In addition, the 60 elderly patients in the trial and control groups will receive clinical visits at the baseline and after 6, 9, and 12 months post-intervention using scales and questionnaires. The primary treatment and intervention outcome evaluation index shows the change in the total Geriatric Depression Scale (GDS) score. A secondary index includes the change in the total Older People Quality Of Life (OPQOL) score, the 36-item short-form (SF-36) from the Medical Outcomes Study (MOS), the Social Disability Screening Schedule (SDSS), the Family APGAR Scale, the Montreal Cognitive Assessment (MoCA), the Knowledge-Attitude-Practice (KAP) for LLD, the General Self-Efficacy Scale (GSES), and the Irritational Beliefs Scale (IBS). That is, the effectiveness of the supportive PDH intervention will be comprehensively evaluated in terms of depressive symptoms, QOL, and social, family, and cognitive functioning. See **Figure 1** for a detailed overview of the research procedure.

## Data Collection

### Screening, Demographics, Depressive Symptoms, Quality of Life, Social Functioning, Family Functioning, and Cognitive Functioning Data

At screening, the trained investigators will examine, gather, and record participant eligibility. The Chinese version of MINI (27) will be used to confirm DSM-IV TR criteria for MDD (28) and assess substance/alcohol abuse disorders and other potential exclusion criteria. After enrollment, to investigate the risk factors of LLD, data will be collected regarding the demographics, lifestyle, medical history, sleep quality, family history of psychiatric disease, LLD course, treatment and intervention process, concurrent treatment and intervention measures, social support, negative life events, activities of daily living, interests and hobbies, local mental health resources, and KAP for LLD, using a set of questionnaires that we will design. Other data, including coping style, personality traits, and the severity of depressive symptoms, will be assessed and acquired using standardized Chinese scales and questionnaires, respectively (29, 30).

Finally, to evaluate the effectiveness of supportive PDH intervention, follow-up data regarding improvements in depressive symptoms, QOL, and social, family, and cognitive functioning will be assessed and acquired using other standardized Chinese scales and questionnaires, respectively (29–31).

### Sample Size, Power, and Effect Size

The primary goals of this study are to analyze the epidemiological characteristics and construct a risk model for LLD in Liaoning. The study design for this part is a cross-sectional study. Wang et al. reported the prevalence of MDD in elderly Chinese at 2.8%, where the ratio of urban to rural was 1.4:1 (10). That is, if the testing level α and the relative error ϵ are set to 0.05 and 0.15P, 6179 community-residing elderly adults are expected to enroll, according to the following equation (32):

$$\text{N}=[Z_{a/2}^{2}P(1-P)]/\epsilon^{2}$$

Given that 10% of the data are missing, a total of 6,800 elderly residents (approximately 3414 urban residents and 3,386 rural residents) should be included.

The secondary goals of this study are to conduct and evaluate integrated supportive PDH interventions and TAR for the objects in the trial group and control group, respectively. The study design for this part is an experimental study, and the sample size can be determined by the change in the value of the mean and standard deviation of the depression score of elderly patients before and after intervention in a previous study (33). That is, if the testing level α and type-II error β are set as 0.05 and 0.10, respectively, the sample size of the trial group and control group will be roughly 25 in both cases, according to the following equation (32):

$${\text{N}}_{1}={\text{N}}_{2}=2[\frac{({\text{z}}_{\alpha /2}+{\text{z}}_{\beta })\sigma }{\sigma }{]}^{2}$$

Given that 20% of the data are missing, each group should have a sample size of 30 LLD patients. Meanwhile, to facilitate the compliance of patients in the later intervention stages, and to ensure the effectiveness of the intervention, each patient’s primary caregiver will be required to accompany the participant. In the later stages, we will further revise the sample size according to the preliminary experimental results from the intervention study.

The participants, method, assessment time, and data collection administrators are listed in **Table 3**.

**Table 3:** Data collection at baseline and follow-up evaluation.

Domain	Measure	Participants	Method	Assessment time	Administrator
Informed consent	Informed consent	All participants ^b^	Interview	Screening	Investigator
Eligibility	Inclusion/Exclusion	All participants ^b^	Interview	Screening	Investigator
Psychiatric diagnoses	MINI	All participants ^b^	Interview	Screening	Investigator
MDS for the elderly ^a^	Team-designed questionnaire	All participants ^b^	Interview	Baseline	Investigator
Coping style, personality traits	SCSQ, EPQ	All participants ^b^	Interview	Baseline	Investigator
KAP for LLD	Team-designed questionnaire	All participants ^b^	Interview	Baseline and every follow-up	Investigator
Local mental health resources and resources available to LLD patients	Team-designed questionnaire	All participants ^b^ and staff of CHS institutions	Interview	Baseline	Investigator
Treatment and intervention process, concurrent treatment and intervention measures	Team-designed questionnaire	All participants ^b^	Interview	Baseline and every follow-up	Investigator
Depressive symptoms	GDS	60 LLD patients and their primary caregivers ^c^	Interview	Baseline and every follow-up	Investigator
Quality of life	SF-36, OPQOL	60 LLD patients and their primary caregivers ^c^	Interview	Baseline and every follow-up	Investigator
Social functioning	SDSS	60 LLD patients and their primary caregivers ^c^	Interview	Baseline and every follow-up	Investigator
Family functioning	Family APGAR Scale	60 LLD patients and their primary caregivers ^c^	Interview	Baseline and every follow-up	Investigator
Cognitive functioning	MoCA, GSES, IBS	60 LLD patients and their primary caregivers ^c^	Interview	Baseline and every follow-up	Investigator

LLD, Late Life Depression; MINI, Mini-International Neuropsychiatric Interview; MDS, Minimum Data Set; SCSQ, Simplified Coping Style Questionnaire; EPQ, Eysenck Personality Questionnaire; KAP, Knowledge-Attitude-Practice; GDS, Geriatric Depression Scale; SF-36, The MOS item Short From Health Survey; OPQOL, Chinese Revised Version of Old People Quality Of Life Questionnaire; SDSS, Social Disability Screening Schedule; APGAR, Adaptability, Partnership, Growth, Affection, and Resolve; GSES, General Self-Efficacy Scale; IBS, Irritational Beliefs Scale; MoCA, Montreal Cognitive Assessment; CHS, Community health service.

a Content of MDS includes demographics, lifestyle, medical history, sleep quality, family history of psychiatric disease and LLD course, social support, negative life events, daily activities, interests, and hobbies.

b “All participants” refers to the 6,800 community-residing elderly adults.

c 30 LLD patients and their primary caregivers in the trial group, and 30 LLD patients and caregivers in the control group.

### Data Preprocessing and Statistical Analysis

The statistical analyses will be performed by employing the SPSS statistical package, version 20.0 (SPSS Inc., Chicago, IL, USA), and all evaluations of significance will be determined based on two-sided tests using the 0.05 level of statistical significance. In addition, missing values will be replaced by the column mean/median in this study. The details of the analytic approach are as follows.

1. Demographic and clinical characteristics, lifestyle patterns, medical history, history of alcohol and drug abuse or dependence, family history of mental illness, self-knowledge and treatment attitude, and the self-efficacy levels and depressive symptoms of the subjects will be described by frequency distribution, percentage, arithmetic mean, and standard deviation.
2. Comparison of the above variables between patients with or without LLD will be made using the independent-sample *t*-test, Mann–Whitney *U*-test, and the chi-squared test, as appropriate.
3. Correlation analysis, logistic regression (for binary variables), and multiple linear regression (ANCOVA for continuous variables) will be used to determine whether the variables can enter SEM, and to test the direct and indirect pathways among these hypothesized determinants.
4. Amos version 22.0 statistical software will be used for SEM of the risk factors for LLD.
5. GDS, SDSS, SF-36, OPQOL, Family APGAR, GSES, IBS, MoCA, and the related KAP for LLD in the elderly will be used as evaluation indices, and analysis of variance (ANOVA) will be applied to compare the results of the control and the trial groups before and after intervention.
6. Regression analysis will be used to analyze the influence of various factors on the effectiveness of the supportive PDH intervention.

### Ethical Considerations

This study [approval no. (2019) 2019-312-2] was approved by the Ethics and Research Committee of the First Affiliated Hospital of China Medical University, which verified that the study will be performed in accordance with all ethical standards set forth by the committee.

Participants will be assured of the confidential nature of the study, and written informed consent will be obtained. All data collection will be anonymous and in accordance with the provision of Chinese law regulating patient autonomy, rights, and responsibilities in the field of clinical information and documentation.

## Discussion

Based on an analysis of the current clinical report results, there are many basic studies and investigations of the pathogenic factors of LLD. Investigations mainly focus on three groups of variables. First, researchers study the correlation between the characteristics (34, 35) (biological factors such as disease course, classification, demographics, etc.) of the disorders and their occurrence and development. Second, there is a focus on psychological factors (36, 37) (personality traits, attribution and coping style, self-esteem, etc.) and how they act on the occurrence and development of the disease. Third, researchers consider the relationship between social factors (38–40) (stressful life events, family environment, social support, marital status, economic income level, etc.) and the occurrence and development of the disease. To some extent, these studies have revealed the relationship between LLD and various influential factors, and they have identified the related variables that can predict the condition and outcome of LLD. However, few studies provide a new perspective for studying the process of integrating multiple influential research variables, while analyzing the path of interaction between variables and exploring the influence of various factors on the overall research. In our study, therefore, biological, psychological, and social factors will be integrated to analyze and predict LLD risk factors. In addition, the results will be incorporated into the system of occurrence and development of the disorder. Then, SEM of the risk factors for LLD will be constructed. Furthermore, in view of differences in the prevalence of LLD between rural and urban areas, participants will be recruited from both rural and urban sites for a broadly inclusive and representative elderly population that ensures the equilibrium of sample sources. In doing so, the results and conclusions of the study will be widely generalizable.

Given the different causes, courses, and clinical manifestations of LLD, the treatment response of antidepressants varies. Evidence from literature reviews on depressive disorders indicates that comprehensive psychosocial intervention can effectively make up for the shortcomings of single drug treatments, while improving therapeutic effect (41). Our study regards community-residing elderly adults with LLD as the research subjects, unlike previous studies, which were restricted to inpatients or discharged patients. Further, we consider supportive psychosocial, drug treatment, and health education interventions to be pertinent, based on the identified risk factors of LLD. The formulation of the content, forms, and methods of interventions will be repeatedly revised by the team members based on literature reviews, expert consultation, and preliminary experimental results. The effectiveness of intervention will be evaluated according to improvements in depressive symptoms, family functioning, social functioning, QOL, and KAP for LLD. An SIHM model for LLD will ultimately be obtained by referring to the connotations and theory of health management. In addition, strict quality control procedures, validated assessments by trained investigators, and appropriate statistical analyses will be designed and applied in this study.

There are three anticipated limitations to this study. First, data collection at the baseline and with follow-ups will be completed through questionnaires and scales sent to elderly residents and their primary caregivers. As such, the results may be affected by the cooperative attitude, understanding, and educational level of the respondents. Second, because questionnaire surveys are limited by the designed items and established form of question-and-answer, the researchers cannot fully understand the respondents’ innermost feelings, thoughts, and opinions on some items beyond what is provided by the questionnaires and scales. For that, quantitative surveys and qualitative interviews might be combined in future studies to evaluate the effect of supportive PDH intervention more thoroughly. Third, the sample source of this study is limited to LLD patients in Liaoning province, so the promotion of the research results on supportive intervention and health management model for LLD patients in communities may be limited. By conducting parallel control studies in multiple regions and increasing the sample size and sources, it can be further validated and supported.

## Conclusions

The prevalence and detection rate of LLD has gradually increased over the past several years, and LLD affects an increasing number of elderly people and their families. However, the disorder remains under-diagnosed and under-treated, especially in Liaoning province, China. To our knowledge, there is no relevant research on the prevalence, incidence, and characteristics of LLD in Liaoning province based on large sample surveys. Identifying the risk factors of LLD and constructing integrated supportive PDH intervention models will assist with the treatment and intervention effectiveness of LLD. The results of this study will provide strong and suitable evidence for constructing a risk factor model of LLD, and this will enhance the effectiveness of supportive PDH intervention for LLD patients in rural and urban communities. Ultimately, this study will construct an SIMH model that integrates risk factor identification, LLD diagnosis, and a formulation and implementation of supportive PDH intervention, along with an evaluation of its effectiveness based on full consideration of the status of mental health resources in Liaoning, LLD characteristics, and the actual situation faced by elderly patients and their families.

## Ethics Statement

The studies involving human participants were reviewed and approved by the Ethics and Research Committee of the First Affiliated Hospital of China Medical University. The patients/participants provided their written informed consent to participate in this study.

## Author Contributions

GZ designed and revised the paper. LD wrote the paper. XS contributed to the coordination between community health services institutions and ethics formalities. CF and CT were responsible for data collection and management.

## Funding

This project was supported by a grant from the Major Project of the Department of Science & Technology of Liaoning Province (2019JH8/10300019) and a grant from the Major Project of the Science and Technology Ministry in China (2017YFC0820200).

## Conflict of Interest

The authors declare that the research was conducted in the absence of any commercial or financial relationships that could be construed as a potential conflict of interest.
